# One-step cathodic electrodeposition of a cobalt hydroxide–graphene nanocomposite and its use as a high performance supercapacitor electrode material

**DOI:** 10.1039/c8ra04105a

**Published:** 2018-07-27

**Authors:** Seyed Abbas Rahimi, Parviz Norouzi, Mohammad Reza Ganjali

**Affiliations:** Center of Excellence in Electrochemistry, Faculty of Chemistry, University of Tehran Tehran Iran norouzi@khayam.ut.ac.ir pnorouzi@gmail.com; Biosensor Research Center, Endocrinology & Metabolism Molecular-Cellular Sciences Institute, Tehran University of Medical Sciences Tehran Iran

## Abstract

In this study, Co(OH)_2_-reduced graphene oxide has been synthesized using a simple and rapid one-step cathodic electrodeposition method in a two electrode system at a constant current density on a stainless steel plate, and then characterized as a supercapacitive material on Ni foam. The composites were characterized by FT-IR, X-ray diffraction, scanning electron microscopy, and cyclic voltammetry using a galvanostatic charge/discharge test. The feeding ratios of the initial components for electrodeposition had a significant effect on the structure and electrochemical performance of the Co(OH)_2_-reduced graphene oxide composite. The results show that the 1 : 4 (w/w) ratio of GO : CoCl_2_·6H_2_O was optimum and produced an intertwined composite structure with impressive supercapacitive behavior. The specific capacitance of the composite was measured to be 734 F g^−1^ at a current density of 1 A g^−1^. Its rate capability was ∼78% at 20 A g^−1^ and its capacitance retention was 95% after 1000 cycles of charge–discharge. Moreover, its average energy density and power density were calculated to be 60.6 W h kg^−1^ and 3208 W kg^−1^, respectively. This green synthesis method enables a rapid and low-cost route for the large scale production of Co(OH)_2_-reduced graphene oxide nanocomposite as an efficient supercapacitor material.

## Abstract 2

In this study, Co(OH)_2_-reduced graphene oxide has been synthesized using a simple and rapid one-step cathodic electrodeposition method in a two electrode system at a constant current density on a stainless steel plate, and then characterized as a supercapacitive material on Ni foam.

## Graphical abstract

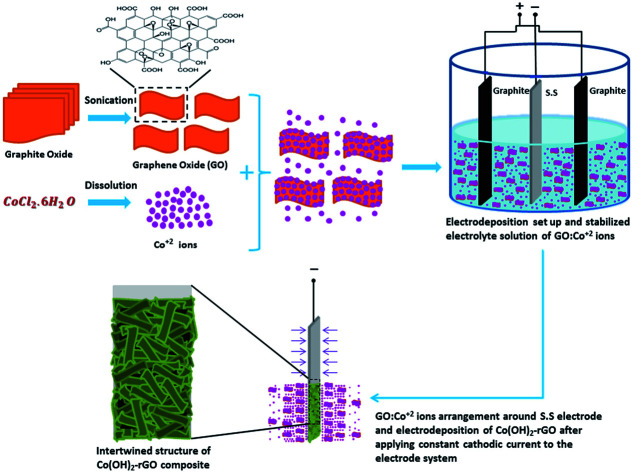

## Introduction

At present, the demand for and application of energy is essential for the development of modern technologies. However, the growing needs for energy bring about environmental problems and the consumption of natural resources. Hence, extreme research for developing new technology on energy storage and delivery has allured much attention.^1–3^ In relation to this, supercapacitors (SCs) are regarded as encouraging candidates for energy storage due to their high power performance, long life cycle, fast charging rate and low maintenance cost.^4–6^ In general, supercapacitors can be classified into two types: (i) electrical double-layer capacitors (EDLCs) that use carbon-based materials as electrode material and (ii) pseudocapacitors that employ transition metal oxides/hydroxides and conductive polymers.^7,8^

Carbon-based EDLCs typically display excellent rate capability and very good cycle lives, but they have low specific capacitances because of the surface accessibility of the carbonaceous material to the electrolyte. Moreover, metal oxides/hydroxides-based pseudocapacitors show high specific capacitances through a fast faradic reaction, but they suffer from low power density arising from weak electrical conductivity of the electroactive material. This restricts fast electron transfer and leads to poor cycling stability because of probable damage to the electroactive material structure during the charge–discharge cycles.^9^ Consequently, applying a hybrid material of carbonaceous materials and metal oxides/hydroxides are expected to have a good performance in supercapacitors.^10,11^

Among a wide variety of metal oxide/hydroxide pseudocapacitive materials (*e.g.* RuO_2_·H_2_O, IrO_2_·H_2_O, MnO_2_·H_2_O, V_2_O_5_, Co_3_O_4_, Co(OH)_2_ and Ni(OH)_2_),^12^ cobalt hydroxide is considered a promising candidate owing to its low cost, well-defined electrochemical reactions, layered structure with large interlayer spacing that facilitate the diffusion of electrolyte ions to the surface of electroactive material, and environmental compatibility.^13–15^ In view of the fact that Co(OH)_2_, as a pseudocapacitive material, has limitations such as low conductivity and intense agglomeration during charge and discharge processes, its application as a high-performance supercapacitor material has not yet been realized.^16,17^ In this regard, many researchers have attempted to overcome these limitations and boost the supercapacitor properties of Co(OH)_2_ by using new methods and strategies for synthesis and modification of individual structures^18–21^ or hybrid structures of Co(OH)_2_.^22–26^ In the same vein, using carbon-based materials in combination with Co(OH)_2_ is one of these strategies to take advantage of synergistic effects and to improve supercapacitor properties of Co(OH)_2_. Among carbonaceous materials, graphene has attracted intense interest because of its unique electrical and mechanical properties, such as high electrical conductivity (10^3^ to 10^4^ S m^−1^) and large specific surface area (calculated theoretical value 2630 m^2^ g^−1^).^27,28^

Recently, cobalt hydroxide-reduced graphene oxide (Co(OH)_2_-rGO) composites have been prepared with different morphologies and structures by different chemical and electrochemical methods. Chemical precipitation,^23,29^ chemical bath deposition,^30,31^ chemical reduction precipitation,^32^ and hydrothermal preparation^33–41^ are examples of chemical methods that have been used to prepare Co(OH)_2_–graphene composites. Also, the preparation of Co(OH)_2_–graphene composites was performed by using electrochemical methods, such as cathodic electrodeposition after Radio Frequency-Plasma Enhanced Chemical Vapor Deposition (RF-PECVD),^42,43^ potentiodynamic electrodeposition^15^ and electrodeposition using chronopotentiometry.^44,45^

Although in the various abovementioned methods some excellent achievements, such as high specific capacitances and long cycle life (1636 F g^−1^,^44^ 1139 F g^−1^,^31^ 1030 F g^−1^,^14^ 960 F g^−1^ ^36^), have been gained, a number of challenges still need to be addressed, such as the complexity of the synthesis method, the expense of equipment and consumption of time. To the best of our knowledge, in only one single report, graphene–Co(OH)_2_ supercapacitor electrode material has been prepared by a simple one-step chemical reduction method.^32^ Although one step electrochemical synthesis of rGO-Ni(OH)_2_ ^46^ and *in situ* glucose-assisted electrodeposition of rGO/MnO_2_ ^47^ has been reported, there is no report yet in the literature on the one-step electrochemical deposition of Co(OH)_2_-rGO nanocomposite.

In the present study, a facile and rapid one-step cathodic electrodeposition method has been developed for the synthesis of Co(OH)_2_-rGO composite. Moreover, this study offers a green preparation method for the composite. These features provide us with an opportunity to have facile control on the synthesis parameters.

The electrochemical properties of the as-prepared supercapacitor electrode materials were investigated in detail. In addition, the effect of the ratio of starter components on the structure and electrochemical properties of the hybrid material is addressed. Moreover, the structural and supercapacitive properties of the as-prepared Co(OH)_2_–graphene composite was compared with that of pure Co(OH)_2_, which was synthesized in a similar way.

## Experimental section

### Chemicals

CoCl_2_·6H_2_O, graphite flakes, KMnO_4_, KOH, H_2_SO_4_ (98%), H_3_PO_4_ (85%), HCl (37%) and H_2_O_2_ (30%) were obtained from Merck chemical Co. Polytetrafluoroethylene (PTFE), *N*-methyl-2-pyrrolidone (NMP) and carbon black were purchased from Sigma-Aldrich Co. Ni foam was purchased from nano-BAZAR Co., Ltd. (Tehran, Iran). All chemicals were used without any further purification and all aqueous solutions were prepared using deionized water.

### Synthesis of graphene oxide (GO)

GO was prepared from graphite flake powder according to the modified Hummer method.^48^ In summary, 1.0 g of graphite powder was added to a 9 : 1 (v/v) mixture of H_2_SO_4_ : H_3_PO_4_. After sonication of the mixture for 1 h, 6.0 g of KMnO_4_ was added slowly (within 5 minutes) with stirring while the temperature was maintained below 40 °C. About 2–3 h after addition of the KMnO_4_, the color of the mixture turned from dark purplish green to dark brown. The reaction mixture was stirred at ∼50 °C for 12 h, following which the solution was cooled to room temperature and then poured onto crushed ice (∼300 mL) with 3 mL H_2_O_2_ to end the reaction. The color of the solution turned bright yellow. The graphene oxide (GO) formed was washed several times with 5% HCl and then with deionized water. The washing treatment was performed using decantation of supernatant by centrifugation. Finally, the gel-like product was dried in vacuum at 80 °C and finally, a dark brown product was obtained.

### Preparation of Co(OH)_2_-rGO nanocomposite by cathodic electrodeposition

GO powder and CoCl_2_·6H_2_O were used to prepare electrolyte solutions for cathodic electrodeposition. Four different weight ratios of these components were used. The following is a typical process for preparation of 1 : 4 (w/w) ratio of GO. CoCl_2_·6H_2_O and 100 mg of GO powder was dispersed in 200 mL deionized water by ultrasonic treatment (at least 1 h). Then, 400 mg of CoCl_2_·6H_2_O was dissolved in ∼50 mL of deionized water and added to the above GO dispersion, which was further diluted to 500 mL and then stirred for 20 minutes to obtain a homogenous mixture.

Cathodic electrodeposition was carried out in a simple two-electrode system containing a stainless steel plate (10 cm × 5 cm) as the cathode electrode centred between two graphite plates (12 cm × 5 cm) as the anode electrodes (at 1 cm space). The cell system was connected to a power supply. The Co(OH)_2_-rGO composites were electrodeposited at a constant current density of 1.7 mA cm^−2^ for 15 min. After electrodeposition, the cathode electrode was washed with deionized water and dried at 60 °C and then, the film was removed from the electrode surface. The obtained samples were composites of 1 : 2, 1 : 4, 1 : 8, and 1 : 20 (w/w) ratios of GO : CoCl_2_·6H_2_O and were labelled as GC2, GC4, GC8, and GC20, respectively. To study the effect of GO, other pure Co(OH)_2_ nanostructures were electrodeposited from a solution containing 400 mg of CoCl_2_·6H_2_O under the same conditions.

Cobalt chloride salt was used as an ion source and supporting electrolyte in the electrochemical system. Then, by applying the constant current to the electrolyte, H_2_O reduction (eqn (1)) and/or the oxygen evolution reaction (eqn (2)) could act as prominent production sources of the hydroxyl group (OH^−^) according to following reactions.^49,50^

1 2H_2_O + 2e^−^ → H_2_ + 2OH^−^, *E*^0^ = −1.08 V

2 O_2_ + 2H_2_O + 4e^−^ → 4OH^−^, *E*^0^ = 0.40 V

In fact, the oxygen evolution reaction is composed of two parallel reactions, eqn (3) and (4), and takes advantage of H_2_O_2_ as an intermediate component to produce OH^−^.

3 O_2_ + 2H_2_O + 2e^−^ → H_2_O_2_ + 2OH^−^

4 H_2_O_2_ + 2e^−^ → 2OH^−^

By applying the cathodic current to the stainless steel electrode, the produced OH^−^ species are localized at the surface of the electrode. As the reaction proceeds, the Co^2+^ ions near the electrode surface undergo hydrolysis and condensation simultaneously and convert to Co(OH)_2_, which and deposits on the electrode based on the following equation:^49,50^

5 Co^2+^ + 2OH^−^ → Co(OH)_2_

**Fig. 1:**
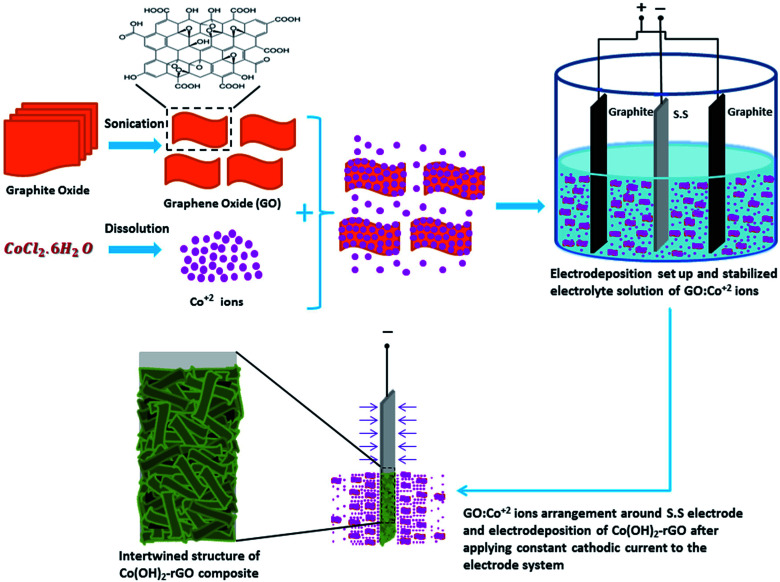
Schematic illustration of the one-step cathodic electrodeposition process of Co(OH)_2_-rGO composite in a simple two electrode system.

In the case of Co(OH)_2_-rGO synthesis, the dispersed sheets of GO in solution are negatively charged due to the existence of the ionized hydroxyl and carboxyl groups on their surface.^51,52^ After the cobalt salt was added to the solution, these functional groups electrostatically absorb the Co^2+^ ions. This phenomenon could allow for the simultaneous hydrolysis and condensation reactions of the Co^2+^ ions with OH^−^ along with co-deposition and reduction of GO sheets to form Co(OH)_2_-rGO on the electrode surface. The schematic picture of cathodic electrodeposition of Co(OH)_2_-rGO is shown in Fig. 1.

### Characterization

The chemical ingredients, morphology and crystalline structure of the electrodeposited samples were investigated by Fourier transform infrared spectroscopy (FT-IR, Perkin Elmer), scanning electron microscopy (SEM, COXEM) (supplied with an energy dispersive spectrometer, EDS, IXRF), and X-ray diffraction (XRD, STOE, provided with Cu-Kα radiation *λ* = 1.54056 Å).

### Electrochemical testing of the composite electrode material

Electrochemical properties of the composites were investigated under a three-electrode electrochemical cell at room temperature. The working electrodes were fabricated by mixing the well-prepared powder of active materials with acetylene black and PTFE in a mass ratio of 85 : 10 : 5 and dispersed in NMP solvent. PTFE and acetylene black were used as the binder and conductive agent, respectively. The as-prepared slurry was coated onto the Ni foam substrate (1 cm × 1 cm) using a spatula and then dried at 60 °C for 12 h. Finally, the electrode was pressed under a pressure of 2 MPa. A platinum wire and an Ag/AgCl electrode were used as the counter and the reference electrode, respectively. The as-prepared electrodes were immersed in 2.0 M KOH solution for at least 8 h. The electrochemical measurements were carried out in a 2.0 M KOH aqueous electrolyte. Cyclic voltammogram (CV) and galvanostatic charge/discharge (GCD) curves were recorded by a potentiostat (Autolab PGSTAT302N). Electrochemical Impedance Spectroscopy (EIS) was recorded at 0.1 V AC voltage within a frequency range of 100 kHz to 0.1 Hz. All electrochemical experiments were performed at room temperature.

## Result and discussion

### Characterization of electrode materials

**Fig. 2:**
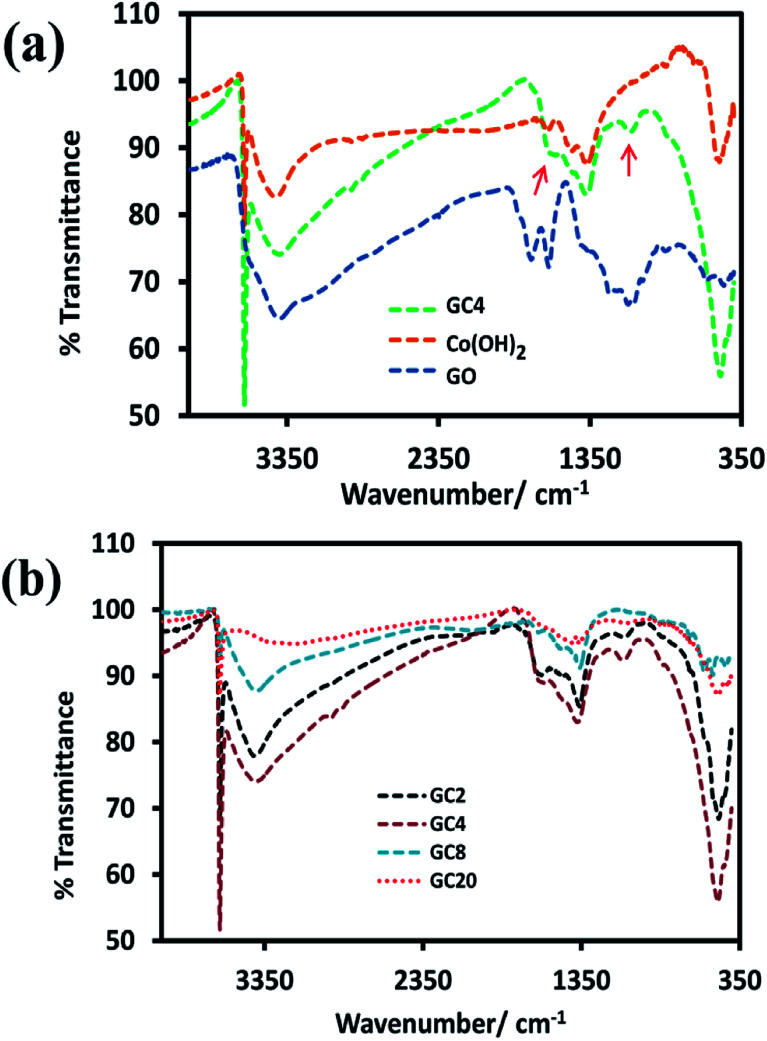
FT-IR spectrums of (a) GO, Co(OH)_2_ and GC4 composite and (b) four Co(OH)_2_-rGO hybrid materials with different weight ratios of initial components (GO and cobalt salt).

For the investigation of functional groups, the FT-IR spectrum of GO and GC4 (as an ideal component ratio based on our studies) and four composites having different weight ratios of GO and Co salt were compared (see Fig. 2(a) and (b)). All FT-IR samples were prepared by mixing the fixed ratio of each sample (2 mg) and KBr (0.4 g) and pressed under pressure.

As shown in Fig. 2(a), in the spectrum of GO, the absorption bands at 3417 cm^−1^ can be assigned to the O–H stretching vibrations. The peak located at 1625 cm^−1^ can be ascribed to the skeletal vibration of C=C from sp^2^ carbon bonds. The peaks located at 1744 cm^−1^, 1389 cm^−1^, and 1230 cm^−1^, can be attributed to the stretching vibrations of C=O in carboxyl groups, C–OH, and C–O–C, respectively.^8,53–56^ Also, the spectra for the GC4 composite and Co(OH)_2_ alone are shown above the GO spectrum. In the spectra of the GC4 composite and Co(OH)_2_, the sharp and intense peak located at 3632 cm^−1^ can be related to the non-hydrogen bonded O–H groups and also indicate the full formation of the β-Co(OH)_2_ phase in both samples.^57,58^ As shown in Fig. 2(a), the intensity of the peak at 3632 cm^−1^, related to β-Co(OH)_2_, is much higher than the intensity of the peak for the Co(OH)_2_ sample, which can be attributed to the synergistic effect of the GO sheets on the phase formation process of Co(OH)_2_ during electrodeposition. Also, the peaks located at 3420 cm^−1^ and 493 cm^−1^ can be attributed to the O–H stretching vibrations of water molecules and the *ν*(Co–O) stretching vibrations, respectively.^32,37^ The intensity of 3632 cm^−1^, 1090 cm^−1^, and 493 cm^−1^ peaks for the GC4 sample are much larger than those seen for the Co(OH)_2_ sample. In addition, two peaks located at 1090 cm^−1^ and 1570 cm^−1^ (marked with red arrows) in the spectrum of GC4 can be assigned to the deformation of the C–O band and the skeletal vibration of the bonded rGO sheets to Co(OH)_2_, respectively.^50,59,60^

According to Fig. 2(b), by comparing the four FT-IR spectra of the composites (synthesized from different weight ratios of the initial components), it can be seen that the intensity of the peaks located at 1090 cm^−1^ and 1570 cm^−1^ (related to rGO) initially increases with the ratio of GO to CoCl_2_·6H_2_O up to 1 : 4 (w/w) and then decreases. The observation of such behavior is probably due to the existence of maximum aggregation at the ratio of 1 : 4 (w/w) *via* electrostatic interactions between cobalt ions and graphene oxide in the as-prepared electrodeposition electrolyte.

**Fig. 3:**
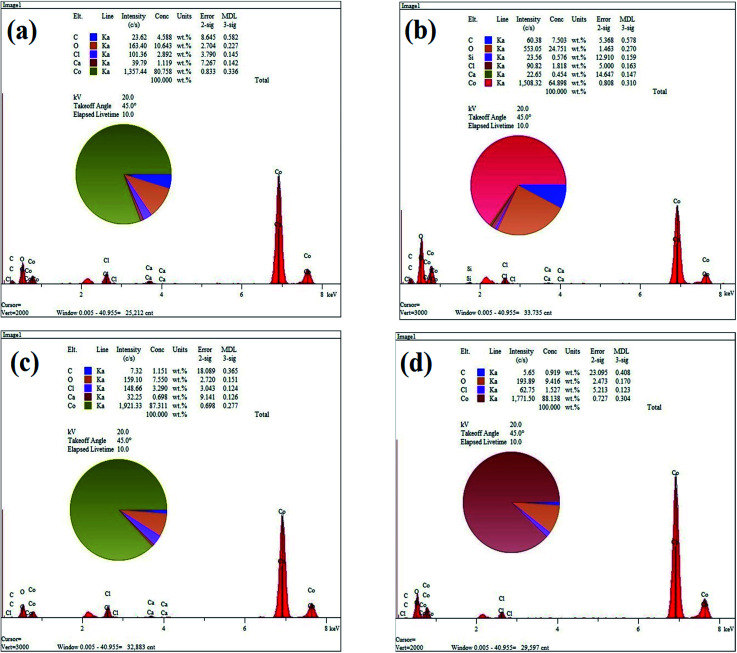
EDS spectrums of (a) GC2, (b) GC4, (c) GC8 and (d) GC20 composite samples.

Fig. 3(a)–(d) show the EDS elemental analysis of GC2, GC4, GC8 and GC20 composites, respectively. The elemental analysis indicates that the carbon content (C%) of the composites is in the following order: GC4 > GC2 > GC8 > GC20. Among these composites, as a result of hybridization of GO with Co(OH)_2_ during the electrodeposition, C% in the GC4 composite is 7.5. This result is in good agreement with the obtained data from the FT-IR spectra (see Fig. 2(b)).

**Fig. 4:**
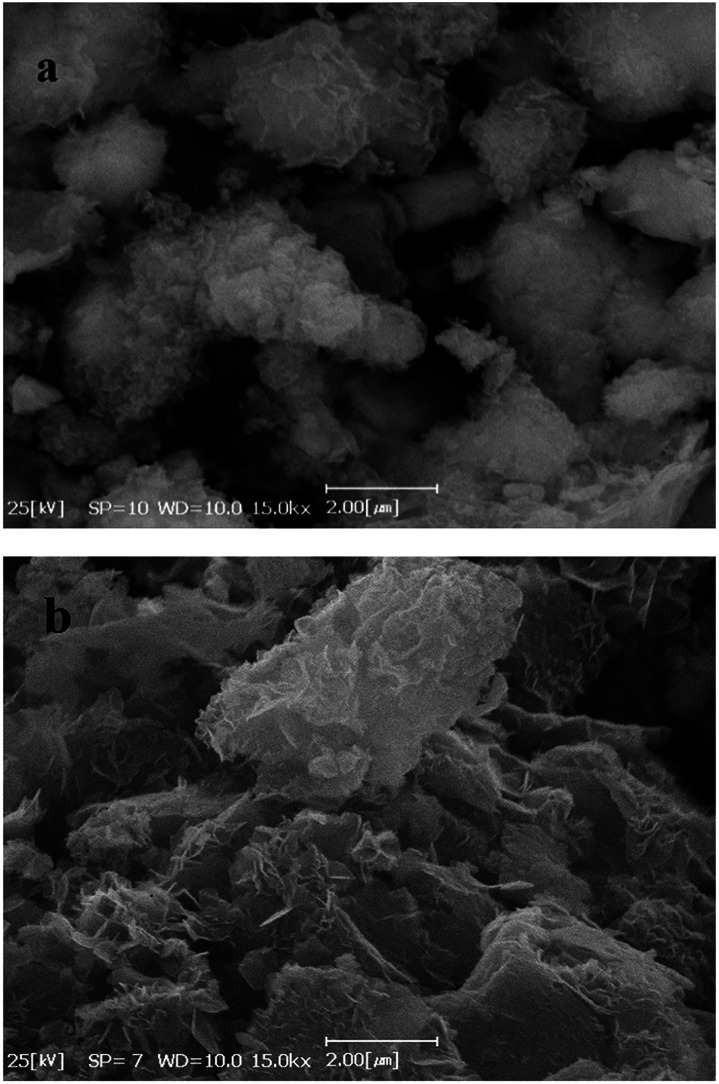
SEM images of (a) Co(OH)_2_, and (b) GC4 composite respectively.

Fig. 4(a) and (b) show SEM images of Co(OH)_2_ and GC4 composite, respectively. On comparison, Co(OH)_2_ exhibits sponge-like structure with some layers that had not been properly completed to form perfect interweave coats, while the GC4 composite displays an intertwined structure with distinct nanostructure sheets. The latter structure shows a well-porous structure that could ideally have more capability to preserve charge.

**Fig. 5:**
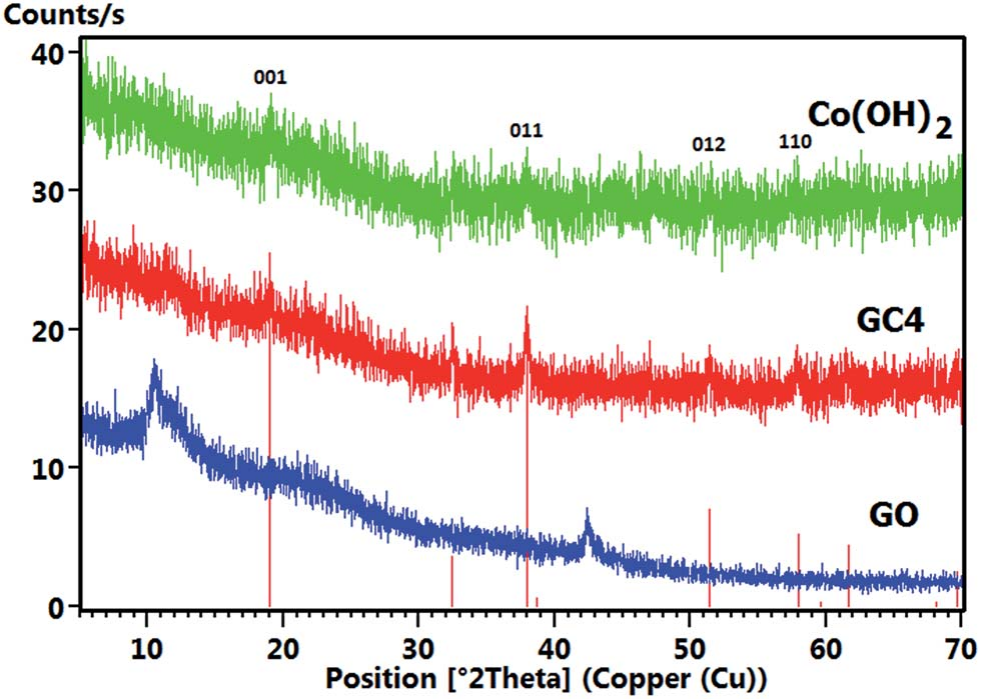
Comparative XRD patterns of the GO, GC4 and Co(OH)_2_ prepared samples.

Fig. 5 shows the comparative XRD patterns of GO, GC4 and Co(OH)_2_. For GO, distinct broad peak at 2*θ* = 10.48° corresponds to diffraction of the (001) plane of the graphene oxide structure with interlayer spacing of 0.84 nm, indicating the somewhat high oxidation degree of graphite.^48^

The XRD pattern of GC4 shows peaks at 2*θ* = 19.11°, 32.56°, 38.03°, 38.78°, 51.51°, 58.10°, 59.74°, 61.75°, 68.20° and 69.71°, which can be respectively indexed as (001), (010), (011), (002), (012), (110), (003), (111), (020) and (103) distinct diffractions of brucite-like β-cobalt hydroxide (ref. code no. 96-101-0268) with hexagonal cell (*a*, *b* = 3.18 + 0.01 Å, *c* = 4.68 + 0.02 Å).^57,58,61^ By comparing XRD patterns of GC4 and Co(OH)_2_, it can be seen that the pattern of Co(OH)_2_ is similar to that of GC4, particularly at (001), (011), (012) and (110) diffractions, but with lower intensity. This can be attributed to the formation of a less crystalline structure of Co(OH)_2_ during electrodeposition in the presence of only Co^2+^ ions and the presence of GO sheets during electrodeposition of GC4, which probably induced better crystalline structure of the composite. The crystallography results from XRD were also confirmed by morphology observations from SEM and by the FT-IR spectra for the chemical bonding (see Fig. 2(a) and 4).

### Electrochemical study

**Fig. 6:**
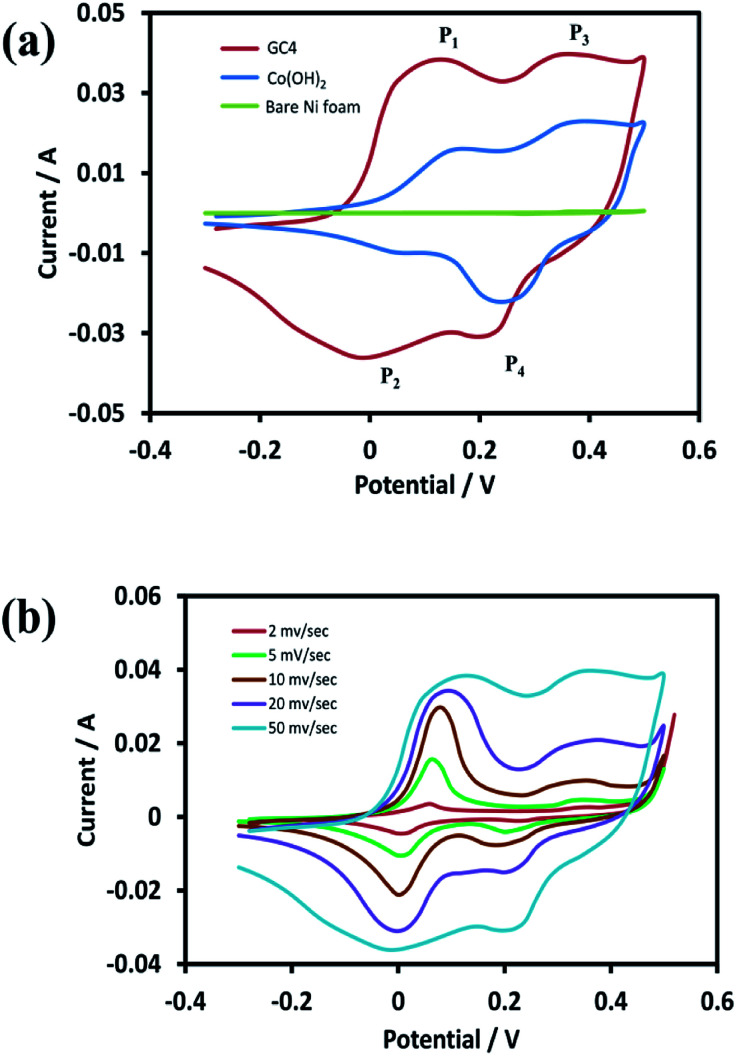
(a) CV curves of GC4 hybrid electrode, Co(OH)_2_ electrode and bare Ni foam at scan rate of 50 mV s^−1^ and (b) CV curves of GC4 at different scan rates in the potential range of −0.3 to 0.5 V.

For the investigation of the electrochemical performance of the electrodeposited materials (as supercapacitors), the as-prepared samples were examined by CV, EIS, and GCD techniques. Fig. 6(a) shows the recorded CVs in the potential range of −0.3 to 0.5 V for the curves of GC4, Co(OH)_2_, and the bare Ni foam at a scan rate of 50 mV s^−1^. As shown in the figure, in case of the bare Ni foam, there was no noticeable peak current. According to the CV curves for the GC4 and Co(OH)_2_ electrodes, a pair of redox peaks for both electrode materials in the KOH electrolyte is observed. The reactions are as follows:^20,24^

6 Co(OH)_2_ + OH^−^ ↔ CoOOH + H_2_O + e^−^

7 CoOOH + OH^−^ ↔ CoO_2_ + H_2_O + e^−^

The electrode reactions during the charging process are related to the oxidation of Co(OH)_2_ to CoOOH with the P_1_ peak potential, followed by the oxidation of CoOOH to CoO_2_ with the P_3_ peak potential. During the discharge process, the reactions correspond to reduction of CoO_2_ to CoOOH and then the reduction to Co(OH)_2_, with the P_4_ and P_2_ peak potentials, respectively.

Taking into account the CV curves data, first, the CV curve profiles of both electrodes exhibit well-defined redox peaks, confirming the faradic nature of the materials. Also, a characteristic of the pseudocapacitive effect can be differentiated from electrical double-layer capacitors, *i.e.*, a typical rectangular shape.^62^ Second, the redox pair peaks are more distinct in the GC4 and appear to have a relatively equal share of total faradic current rather than that seen in case of Co(OH)_2_. This can be attributed to the presence of graphene in the composite structure that has a synergistic interaction with Co(OH)_2_ and hence, it can improve the appearance of the P_1_/P_2_ redox pair peaks.

Fig. 6(b) represents the CV curves of GC4 at different scan rates (2, 5, 10, 20, and 50 mV s^−1^). As seen in the curves, the shapes of the CVs changed with scan rate, which is clearly distinct from that of electrochemical double-layer capacitance that has a rectangular shape. This again confirms the pseudocapacitance behavior for the GC4 composite, in which the charge storage is based on faradic redox reactions (eqn (6) and (7)).^23,40^ Furthermore, the peak current of anodic oxidations is almost equal to that of cathodic reductions. This could be interpreted as good charge–discharge reversibility for the GC4 composite.

**Fig. 7:**
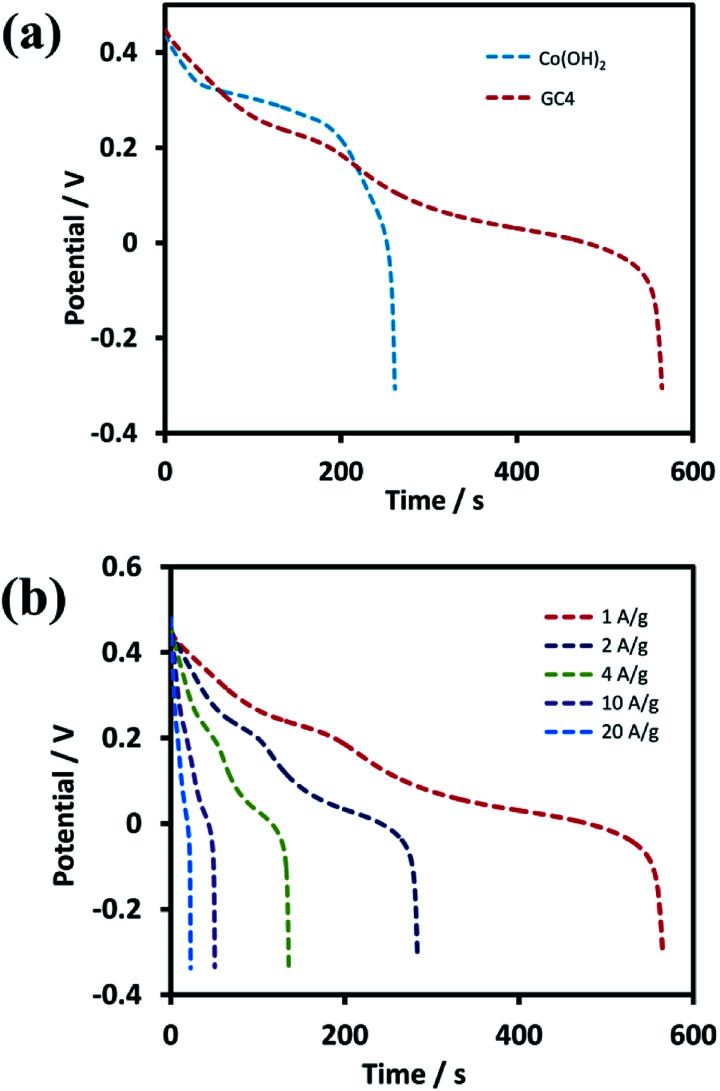
GCD test profiles of (a) GC4 and Co(OH)_2_ electrode within the potential of −0.3 to 0.5 V at current density of 1 A g^−1^ and (b) discharge curves of GC4 hybrid material at different current densities from 1 to 20 A g^−1^.

Further investigations were performed on GC4 and Co(OH)_2_ samples from the GCD experiments. Fig. 7(a) shows the discharge profiles of the as-prepared GC4 and Co(OH)_2_ electrodes within the potential range of −0.3 to 0.5 V at the current density of 1 A g^−1^. The nonlinear shape of the discharge profiles for both samples show pseudocapacitive behavior. The results are also in good agreement with their CV data.^62^ The appearance of two distinct potential plateaus (corresponding to two-steps of reduction of CoO_2_ to Co(OH)_2_) for GC4 in comparison with Co(OH)_2_ implies that the presence of rGO in the hybrid electrode probably causes an increase in the reversibility of the two redox reactions *via* intensifying the bulk conductivity of the composite and facilitating ion diffusion of the electrolyte into the surface of the electrode, which induces the synergistic effect of cobalt hydroxide. Therefore, a boosted supercapacitive performance for the GC4 hybrid material is observed.

Specific capacitances (*C*_s_) of electrodes were measured to be 734 and 352 F g^−1^ at the current density of 1 A g^−1^ for the GC4 and Co(OH)_2_, respectively. The calculation of specific capacitances of electrodes was carried out based on the following equation:^21^8
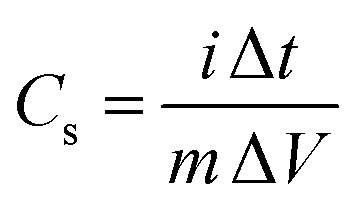
where *C*_s_ (F g^−1^) is the specific capacitance, *i* (A) is the charge–discharge current, *m* (g) is the mass of electroactive material on Ni foam, Δ*V* (V) is the potential drop during discharge and Δ*t* (s) is the discharge time. According to above equation and discharge profiles (not shown here for all composites), the specific capacitances for GC2, GC4, GC8 and GC20 composite electrode materials were calculated to be 615, 734, 490 and 440 F g^−1^ at 1 A g^−1^, respectively. This result shows that the GC4 composite with 7.5% C loading, arising from rGO in the composite structure, has the highest *C*_s_ than that of other samples. Moreover, by comparing specific capacitances for Co(OH)_2_ and GC20 at 1 A g^−1^, we can imply that even with a low level of C loading in the composite structure (see Fig. 3(d)), we can significantly improve charge storage capacity of the GC20 composite.

Discharge curves of GC4 hybrid material at different current densities from 1 to 20 A g^−1^ are shown in Fig. 7(b). The *C*_s_ values of GC4, according to eqn (8), were calculated to be 734, 728, 710, 669 and 570 F g^−1^ at current densities of 1, 2, 4, 10, and 20 A g^−1^, respectively, while the calculated *C*_s_ values of Co(OH)_2_ are 352, 340, 305, 261 and 158 F g^−1^ at current densities of 1, 2, 4, 10 and 20 A g^−1^, respectively. At both electrodes, at high current densities, the *C*_s_ values decreased due to the minimum time for the active material to respond and less access of surface active sites to charge storage due to the blocking effect of the OH^−^ ions diffusion process.^34,63^ The *C*_s_ values show that at the current density of 20 A g^−1^, the rate capability for GC4 and Co(OH)_2_ is as high as ∼78% and ∼55%, respectively. The better rate capability for the GC4 over Co(OH)_2_ implies that the presence of rGO in the composite network provides better diffusion rate of the OH^−^ ions to the surface of electroactive materials even at high current density.

In addition, the energy density (*E*) and power density (*P*) for both Co(OH)_2_ and GC4 electrodes materials were calculated according to eqn (9) and (10).^19,64^9
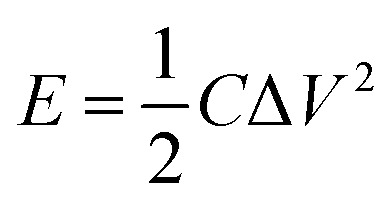
10
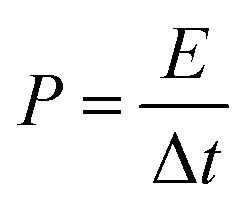


The calculated average values for the energy density and power density at different current densities (1, 2, 4, 10 and 20 A g^−1^) for GC4 were 60.6 W h kg^−1^ and 3208 W kg^−1^, respectively. The calculated *E* and *P* quantities for Co(OH)_2_ were 25.5 W h kg^−1^ and 2575 W kg^−1^, respectively. Again, these values confirm the improvement in supercapacitive performance of the GC4 composite electrode as compared to that of Co(OH)_2_ due to the rGO insertion into the composite.

**Fig. 8:**
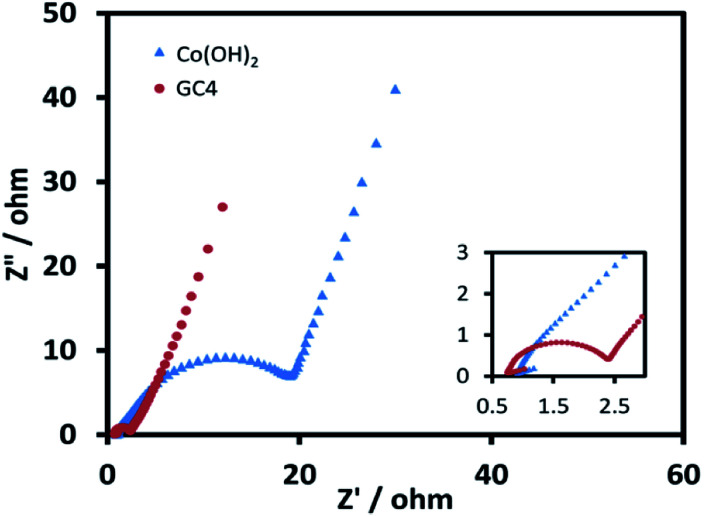
Nyquist plots for GC4 and Co(OH)_2_ electrodes over the frequency range from 100 kHz to 0.1 Hz at 0.1 V.

EIS measurements, as a principal method to evaluate the basic behavior of supercapacitor materials, were conducted in 2 M KOH solution. Fig. 8 shows the Nyquist plots for both electrodes over the frequency range from 100 kHz to 0.01 Hz at 0.1 V. The impedance spectra of both electrode materials are almost similar, and consist of a semicircle at the high frequency region and a post-semicircle straight line at the low frequency region.

The solution resistance (*R*_s_) can be derived from the intercept of the plot on the real impedance axis. The calculated *R*_s_ value for GC4 is 0.74 Ω, which is smaller than that of Co(OH)_2_ (0.85 Ω) and implies that GC4 has good conductivity. In the high frequency region, the diameter of the semicircle represents the charge transfer resistance (*R*_ct_) corresponding to the faradic redox reaction of electrode materials, involving the exchange of OH^−^ ions. The calculated quantity of *R*_ct_ for GC4 is 0.82 Ω is much smaller than that of Co(OH)_2_ (9.2 Ω), hinting that the bulk conductivity and response rate of charge transfer in the composite are very favorable. Therefore, GC4 can be considered to be used as an ideal supercapacitive electrode material.^34,63^ The post-semicircle straight line in the low frequency region is known as Warburg resistance, which occurs due to diffusion and transport of the OH^−^ ions in the electrolyte to the electrode surface.^36^ In this region, a line with higher slope than 45° represents lower diffusion resistance to ions.^62^ As shown in Fig. 8, the linear slope for both GC4 and Co(OH)_2_ is apparently greater than 45°, suggesting that diffusion resistance is not the determining factor and hence, both electrodes can store charge effectively.^18,36^

**Fig. 9:**
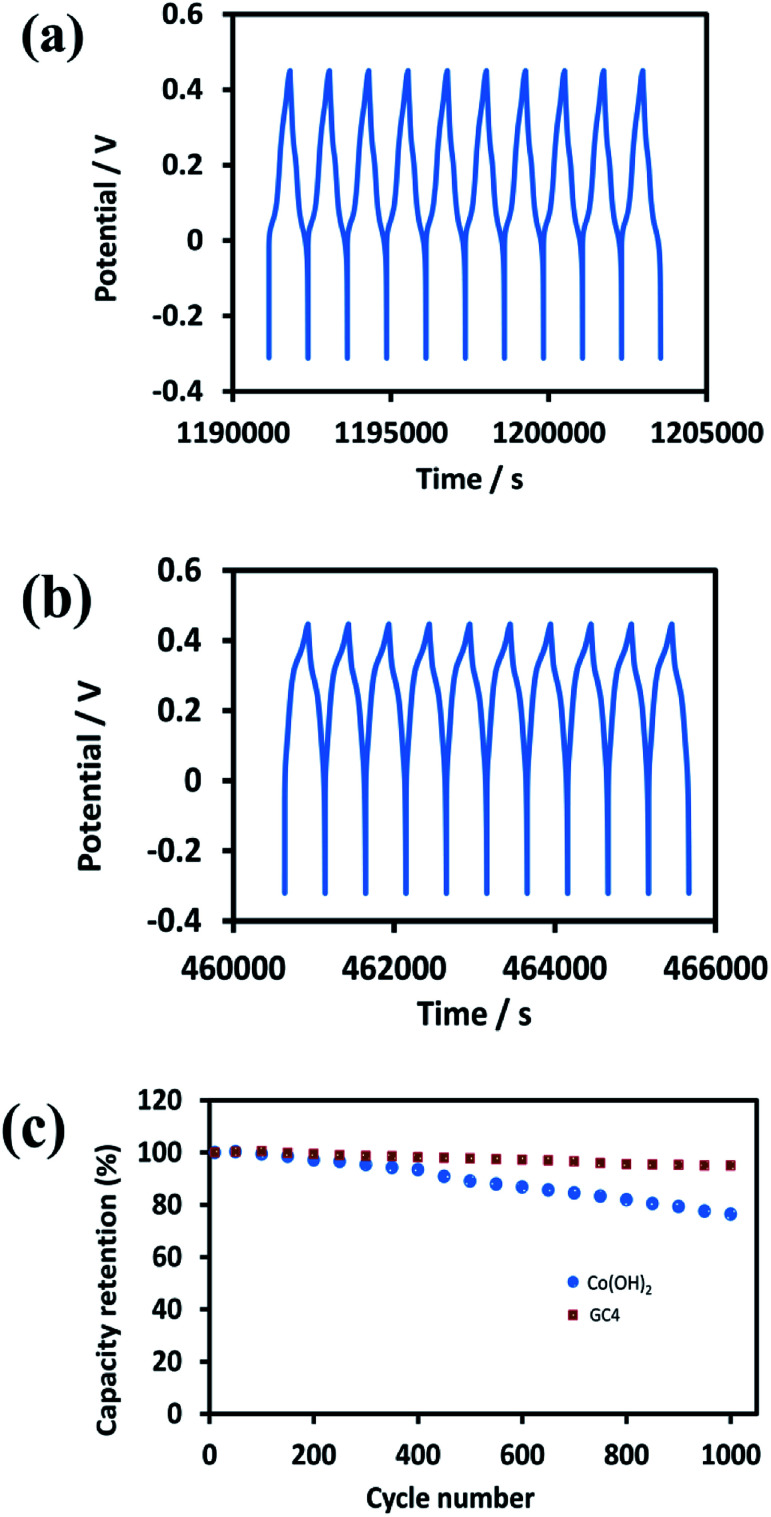
The 10 last cycles of (a) GC4 hybrid electrode (b) Co(OH)_2_ electrode and (c) the cycle stability of two electrode material at current density of 1 A g^−1^ for 1000 cycles.

Good cycle life is a crucial parameter for supercapacitor electrode materials. Fig. 9(a) and (b) represent the 10 last charge–discharge cycles of the composite and Co(OH)_2_ at a current density of 1 A g^−1^, respectively. Fig. 9(c) displays the comparison between the capacitance retention of the two electrode materials over 1000 cycles, estimated from the GCD test conducted at the current density of 1 A g^−1^.

The calculated capacitance retention for GC4 was 95% after 1000 cycles of charge–discharge, while this quantity for pure Co(OH)_2_ was 76%, demonstrating that the GC4 has a long cycling stability and a high degree of charge–discharge reversibility. The improved capacitive performance for GC4 over pure Co(OH)_2_ could be attributed to the intertwined structure of the composite that resulted in more flexibility of the nanostructure sheets of the composite during charge–discharge cycles.

## Conclusions

Co(OH)_2_-rGO hybrid materials were prepared by a one-step cathodic electrodeposition method. Results showed that the 1 : 4 (w/w) ratio of GO : Co salt was optimum for the electrodeposition of the composite (*i.e.* GC4) with the most effective structure. The GC4 composite exhibited a specific capacitance of 734 F g^−1^ at a current density of 1 A g^−1^, a rate capability of ∼78% at a current density of 20 A g^−1^, and a cycling stability of 95% after 1000 cycles of charge–discharge. Also, this composite demonstrated the average energy and power density of 60.6 W h kg^−1^ and 3208 W kg^−1^, respectively. Results exhibited that the boosted supercapacitive performance for the GC4 as compared to that of other composites may be attributed to the synergistic effect between Co(OH)_2_ nanostructure and rGO sheets and the intertwined structure of this composite. Briefly, these results indicate that this study offers an opportunity for rapid and environmentally friendly preparation route of the metal oxide/hydroxide-rGO composites.

Furthermore, we can predict that this procedure can also be used to prepare similar composites by using supportive reagents in an electrodeposition solution for more rGO insertion into the hybrid matrix, and thus realize the more possible improvement in the supercapacitive properties of the composite.

## Conflicts of interest

There are no conflicts to declare.

## Supplementary Material
